# Anticancer Effect of Cisplatin-Loaded Poly (Butylcyanoacrylate) Nanoparticles on A172 Brain Cancer Cells Line

**DOI:** 10.31557/APJCP.2020.21.2.273

**Published:** 2020

**Authors:** Asad Vaisi-Raygani, Mozafar Khazaei, Mitra Bakhtiari, Elham Arkan, Faranak Aghaz

**Affiliations:** Fertility and Infertility Research Center, Kermanshah University of Medical Sciences, Kermanshah, Iran,

**Dear Editor**

Recently, we read with interest the valuable article entitled “Anticancer Effect of Cisplatin-Loaded Poly (Butylcyanoacrylate) Nanoparticles on A172 Brain Cancer Cells Line” that has been published in Asian Pacific Journal of Cancer Prevention (Chiani et al., 2019). The paper has pointed out that cisplatin loaded on poly (Butylcyanoacrylate) nanoparticles with this range of size, was more efficient than the free form of cisplatin in treating A172 cancer cell line that helps to elucidate the role and the mechanism of antitumor activity of NC- cisplatin. 

Among the most important findings, were nessesery several comments to complete, that the authors have ignored them in their article:

1) The nano-carrier stability of cisplatin loaded on poly (Butylcyanoacrylate) nanoparticles could monitor the time-dependent changes in particle size and drug content. To test the chemical stability, the content of Cisplatin loaded on poly (Butylcyanoacrylate) nanoparticles in sample could be determined via high-performance liquid chromatography (HPLC) (Bernabeu et al., 2019). 

2) In vitro cellular uptake of the free and Cisplatin loaded on poly (Butylcyanoacrylate) nanoparticles could be determined by HPLC from lysates of cancer cells (Ferreira et al., 2018; Ferreira et al., 2019).

3) Eke, the morphology measuring of nano-capsulated need be analyzed by a more accurate method, such as microscopic electron techniques (transmission electron microscopy (TEM), scanning electron microcopy (SEM)) that this is a neccessery as a charectristic drug-loaded nanocapsules (Motiei et al., 2017). 

4) The DAPI staining could examine the cell nucleus morphology in normal and apoptotic cells by fluorescence microscopy and the percentage of the apoptotic cells can be estimated by annexin V/propidium iodide staining that will be helpful.

5) Finally, migration ability of the cells can be measured using a wound-healing assay. 

## Conflict of interest

Asad Vaisi-Raygani, Mozafar Khazaei, Mitra Bakhtiari, Elham Arkan, and Faranak Aghaz declare that they have no conflict of interest. 
