# Supplementary material for: Anticancer Effect of Cisplatin-Loaded Poly (Butylcyanoacrylate) Nanoparticles on A172 Brain Cancer Cells Line
Source: Asian Pac J Cancer Prev. 2020;21(2):273–4. doi: 10.31557/APJCP.2020.21.2.273 (PMC7332141; doi:10.31557/APJCP.2020.21.2.273)
Supplement: Related texts

# Authors’ Reply

**Published:** 2020

**Authors:** Mohsen Chiani, Attabak Toofani Milani, Mahdieh Nemati, Jalal Rezaeidian, Hossein Ehsanbakhsh, Zohre Ahmadi, Ebrahim Mazloomi, Vahideh Sadeghi, Azim Akbarzadeh Khiyavi

**Affiliations:** 1 *1Department of Nanobiotechnology, Pasteur Institute of Iran, *; 2 *5Department of Biology, Science and Research Branch, *; 3 *8Department of Genetics, Tehran Medical Sciences Branch, Islamic Azad University, Tehran, *; 4 *2Department of Biochemistry, Faculty of Medicine, *; 5 *7Cellular and Molecular Research Center, Cellular and Molecular Medicine Institute, Urmia University of Medical Sciences, Urmia, *; 6 *3Department of Medical Nanotechnology, Faculty of Advanced Medical Science, Tabriz University of Medical Sciences, Tabriz, *; 7 *4Department of Biology, Parand Branch, Islamic Azad University, Parand, *; 8 *6Faculty of Health, Qom University of Medical Sciences, Qom, Iran. *

**Dear Editor**

In response to very useful and stand comments of the letter regarding our paper titled “Anticancer Effect of Cisplatin-Loaded Poly (Butylcyanoacrylate) Nanoparticles on A172 Brain Cancer Cells Line”. 

 We have to say that, the focus of this paper was mainly on the anticancer activity of Cis-NPs and we would have looked at other properties such as time- dependent stability and NPs morphology or other experiments however, due to limited resources the possibility was none. We do also recommend in future research such properties should be address provide enough resources is available.
